# Multidimensional feature fusion in longitudinal physical examination data: a machine learning framework for classification of type 2 diabetes

**DOI:** 10.3389/fpubh.2026.1841719

**Published:** 2026-08-11

**Authors:** Yan Zhou, Bijun Sun, Xiang Cai, Jiandong Qi

**Affiliations:** 1 Department of General Practice, Beijing Hospital, National Center of Gerontology, National Clinical Research Center for Gerontology, The Key Laboratory of Geriatrics of NHC, Institute of Geriatric Medicine, Chinese Academy of Medical Sciences, Beijing, China; 2 School of Information Science and Technology, School of Artificial Intelligence, Beijing Forestry University, Beijing, China

**Keywords:** intelligent medicine, longitudinal study, multi-dimensional feature extraction and fusion, physical examination data series, type 2 diabetes

## Abstract

**Background:**

Machine learning-based risk classification of type 2 diabetes mellitus (T2DM) has become a prevailing research direction in intelligent healthcare. Nevertheless, current studies suffer from two key limitations. First, most existing models are built on cross-sectional data and cannot capture the dynamic progression characteristics of T2DM. Second, traditional longitudinal time-series models are commonly adopted without sufficiently exploring the deep interactive relationships among multi-temporal physical examination features, limiting model predictive performance.

**Methods:**

To tackle the above drawbacks, this study proposes a novel multi-dimensional feature extraction and fusion network (MFFNet) for T2DM early risk prediction based on sequential annual physical examination data. The proposed architecture comprehensively mines latent feature information from three complementary dimensions: the horizontal interaction of multiple examination indicators within a single period, the longitudinal dynamic evolution pattern of individual indicators across consecutive years, and the cross-temporal synergistic correlations among different indicators across multiple periods. Experiments were conducted on a real-world longitudinal dataset consisting of three consecutive years of physical examination records from the Health Management Center of Beijing Hospital, which presents an extremely imbalanced sample distribution with a positive–negative ratio of 1:8.34.

**Results:**

Comparative experiments with state-of-the-art time-series models (RNN, LSTM, GRU, and Transformer) demonstrate the superiority of MFFNet. The proposed model achieved a sensitivity of 0.8766, a specificity of 0.7082, a negative predictive value (NPV) of 0.9799, and a PR-AUC of 0.4322. Furthermore, SHAP interpretability analysis identified core predictive features for T2DM risk assessment, including age, creatinine (Cr), waist circumference (WC), alanine aminotransferase (ALT), triglycerides (TG), and systolic blood pressure (SBP).

**Conclusion:**

The proposed MFFNet can effectively implement accurate T2DM classification and early risk prediction using longitudinal physical examination sequences. This multi-dimensional feature fusion strategy substantially improves the mining capability of temporal healthcare data. The lightweight and cost-effective MFFNet provides a reliable artificial intelligence-assisted solution for large-scale early pre-screening and risk intervention of T2DM in primary clinical healthcare.

## 1 Introduction

The global prevalence of type 2 diabetes mellitus (T2DM) has reached pandemic proportions, driven by sedentary lifestyles characterized by excessive sugar and fat intake, physical inactivity, and prolonged sitting behaviors. As a metabolic disorder rooted in insulin resistance or pancreatic *β*-cell dysfunction, T2DM not only compromises patients’ quality of life but also induces life-threatening multisystem complications, including cardiovascular disease, diabetic nephropathy, retinopathy, and peripheral neuropathy. These complications are progressively irreversible and associated with high disability rates, imposing a substantial financial burden on healthcare systems and families (1).

China currently bears the most severe diabetes epidemic, it is reported that the diabetic population exceeded 140 million in 2021, with projections reaching 174 million by 2045 (2). To address this situation, innovative diagnostic approaches are urgently needed. Current diabetes screening primarily relies on fasting blood glucose (FBG) or glycated hemoglobin (HbA1c) measurement, as well as oral glucose tolerance tests (OGTT), which require professional medical personnel for operation and incur high costs, posing significant challenges for large-scale implementation in under-resourced areas. Artificial intelligence (AI) leverages cutting-edge algorithms such as deep learning, multimodal fusion, and temporal analysis to deeply mine the value of medical data, including electronic health records, medical images, and follow-up physical examination records. It enables efficient empowerment across the entire clinical workflow, including early disease risk screening (3, 4), computer-aided clinical diagnosis (5–7), prognostic prediction (8), chronic disease management (9), and public health early warning (10), thereby effectively improving the quality of medical services and the efficiency of resource allocation. Scientists are actively exploring AI models for T2DM detection leveraging health big data, with two predominant research pathways: The one is the cross-sectional studies, which built a static risk assessment model through current physiological indicators (such as BMI, blood pressure and blood lipids) (11); The second is the longitudinal cohort studies, which uses sequential medical records (such as electronic health records or laboratory test sequences) to mine the dynamic disease progression patterns (12).

Cross-sectional studies for T2DM detection classify patients’ current diabetic status based on static physiological and demographic indicators. Most existing research employs classical machine learning (ML) methods within this paradigm, typically comparing multiple algorithms to identify optimal performance (13–16). Shaukat et al. (14) evaluated six ML models, including decision trees (DT), k-nearest neighbors (kNN), random forests (RF), Naïve Bayes (NB), logistic regression (LR), and su pport vector machines (SVM), on the PIMA diabetes dataset, and pointed out that LR model achieved the highest accuracy (0.81). Tigga et al. (15) employed machine learning algorithms (including LR, kNN, SVM, Naïve Bayes and DT) to predict the T2DM risk based on 18 questions related to health, lifestyle, and family background. The same algorithms were also applied to the PIMA database. They found that the RF classifier was the most accurate on these two datasets. Meanwhile, Ensemble learning methods have also been widely applied in static prediction for T2DM. By combining multiple weak machine learning models, ensemble learning constructs a robust model to enhance overall prediction accuracy (17–21). Fitriyani et al. (20) designed an ensemble model by integrating multi-layer perceptron (MLP), SVM, DT, and LR. By combining iForest method for outlier eliminatioin and SMOTETomek method for data balancing, the ensemble model achieved 96.74% accuracy and an AUC close to 1, outperforming traditional single-model approach hes. Fazakis et al. (21) proposed a weighted voting ensemble model (WeightedVotingLRRFs) by optimizing RF and LR weights using the genetic algorithm NSGA-II for multi-objective optimization (maximizing AUC and sensitivity). On the ELSA dataset, WeightedVotingLRRFs achieved an AUC of 0.884 and a sensitivity of 85.6%, significantly outperforming risk score systems (e.g., FINDRISC, Leicester) and standalone ML models. Neural networks demonstrate superiority over traditional machine learning methods in feature extraction and classification. More and more researchers are exploring the adoption of neural networks for diabetes prediction (22–26). Madan et al. (23) evaluated five neural architectures, including CNN, DNN, CNN-LSTM, Bi-LSTM, and CNN-Bi-LSTM, on the PIMA Dataset. They adopted ten-fold cross- validation method to evaluate these models, their proposed CNN-Bi-LSTM model achieved superior performance with accuracy (0.98), sensitivity (0.97), and specificity (0.98). Zhao et al. (24) proposed a novel attention-oriented convolutional neural network (SECNN) through the channel attention mechanism, attaining 94.12% accuracy on the NHANES dataset and 89.47% on the PIMA dataset, outperforming traditional machine learning models.

Although cross-sectional studies for T2DM classification offer certain clinical value, their limitations remain significant. First, cross-sectional data only reflect health status at a single timepoint, failing to reveal the dynamic evolution of risk factors over time. This limitation hinders models from accurately identifying patterns of disease onset and progression. Second, single-timepoint data collection risks information incompleteness or inaccuracies, which directly compromise the model’s generalization. To date, no consensus has been reached regarding the optimal choice of T2DM risk assessment models, the variability in predictive performance across models primarily stems from three key factors: (1) data quality and sample size — larger datasets with rigorous preprocessing typically yield higher prediction accuracy; (2) feature engineering efficacy—Incorporating diabetes-specific biomarkers significantly enhances model performance by capturing pathophysiological mechanisms; and (3) latent pattern detection capacity — the differing abilities of models to uncover latent association patterns within static datasets.

Longitudinal cohort studies for T2DM classification leverage continuous multi-year health records and time series models to forecast current or future diabetes status. Key temporal models include RNN (27), LSTM (28), GRU (29), and their variants. These architectures excel at capturing temporal dependencies, enabling dynamic modeling of T2DM progression and improving prediction accuracy and reliability (30). Xiao et al. (31) developed an Interpretable Temporal Neural Turing Machine (ITS-NTM) to predict the prognosis of patients with T2DM by analyzing dynamic changes in clinical and behavioral indicators. Their study evaluated model performance using clinical and behavioral data at baseline and subsequent 3rd, 6th, 9th, and 12th months. Through dynamic prediction with incremental incorporation of subsequent months’ data, the model achieved 92.0% accuracy and 91.8% F1-score when data at 12th month was added. Results demonstrated that predictive performance was improved with the accumulation of temporal data, and longitudinal features was a major influencing factor in the prediction model. Deberneh et al. (32) predicted T2DM occurrence by using electronic health records from 2013 to 2018 collected at a private medical institute in Seoul, they conducted experiments by increasing the number of years used to train the prediction models. The number of years ranged from 1 year (Y) to 4 years (Y, Y-1, Y-2, Y-3). The results show that as the number of years increased, the accuracy of the models also increased. Ravaut et al. (33) used the Ontario Diabetes Dataset (ODD) to identify individuals with diabetes. They selected data sequences within observation windows of 1, 3, 5, and 10 years as inputs to predict whether the individuals were to have an onset of diabetes in a future buffer period ranging from 1 to 10 years. They found XGBoost achieved optimal predictive performance with a 5-year observation window and 1-year buffer period, yielding an AUC of 80.3.

Although longitudinal cohort studies for T2DM prediction reflect the dynamic process of diabetes onset and progression, thereby enhancing prediction accuracy and reliability, this approach still faces challenges. On one hand, current research commonly encounters the scarcity of high-quality, long-term time-series data, prediction based on continuous health monitoring remains in an exploratory phase. Second, sequential data not only encompasses rich health feature information but also contains complex dynamic evolution patterns of critical risk factors, it is very difficult to fully extract and utilize these insights. Most longitudinal cohort studies on T2DM prediction only involve simple applications of classical time-series models, without in-depth exploration of how to accurately extract and utilize effective information from sequence data.

As awareness of health management among Chinese residents improves and the physical examination system matures, constructing longitudinal health profiles for individuals based on annual physical examination data has gradually become feasible. From 2011 to 2020, China’s health examination coverage rate steadily increased, reaching 30% of the total population (34). These standardized and longitudinal physical examination data provide critical opportunities to uncover the early risk trajectories of T2DM. By deeply analyzing the temporal evolution patterns of key indicators in physical examination data and establishing dynamic predictive models, we can achieve personalized health risk assessment, and offer forward-looking decision support for disease prevention and control systems.

This paper proposes a novel T2DM prediction model that innovatively extracts and integrates information from three dimensions within physical examination data sequences: (1) cross-sectional interactions among concurrent indicators (horizontal dimension); (2) dynamic temporal evolution patterns of individual indicators over time (longitudinal dimension); and (3) cross-correlations between different indicators across varying time periods (cross-dimensional). By extracting and integrating these multi-dimensional information, the model overcomes the limitations of traditional single-dimensional analyses, significantly enhancing T2DM prediction accuracy. This approach provides intelligent decision support for early diabetes screening, driving a paradigm shift in disease management from reactive treatment to proactive prevention.

## 2 Methods

### 2.1 Dataset

The physical examination data utilized in this study came from the Health Management Center of Beijing hospital in Beijing, China. Data collection spanned from 2021 to 2024, encompassing 50,080 entries from 16,653 individuals. Among them, 7,897 were female, 8,877 were male, 4,701 were under 40 years old (accounting for 28%), 7,576 were between 40 and 60 years old (accounting for 45.2%), and 4,497 were over 60 years old (accounting for 26.8%). All data are de-identified and meets all privacy requirements in place.

The dataset comprises three categories of indicators: demographic indicators, physical examination indicators, and derived indicators. Demographic indicators include Age, Gender, Smoking History (SH), Alcohol History (AH), and Exercise Frequency (Ex.freq., times/week). Physical examination indicators include High-Density Lipoprotein Cholesterol (HDL-C), Low-Density Lipoprotein Cholesterol (LDL-C), Diastolic Blood Pressure (DBP), Systolic Blood Pressure (SBP), Fasting Blood Glucose (FBG), Hemoglobin A1c (HbA1c), Triglycerides (TG), Hip Circumference (HC), Waist Circumference (WC), Creatinine (Cr), Neutrophil count (NEU), Lymphocyte count (LYM), White Blood Cell count (WBC), Uric Acid (UA), Electrocardiogram (ECG), Height (Ht), Platelet count (PLT), Monocyte count (MONO), Blood Urea Nitrogen (BUN), Alanine Aminotransferase (ALT), Weight (Wt), Heart Rate (HR), Eosinophil count (EOS), Red Blood Cell Distribution Width (RDW), Basophil count (BASO), Platelet-Large Cell Ratio (P-LCR), Mean Corpuscular Volume (MCV), Mean Corpuscular Hemoglobin (MCH). Derived indicators refer to those identified through literature review as significantly impacting diabetes prediction and calculable from physical examination indicators, including Body Mass Index (BMI) and waist-to-height ratio (WHtR). All indicators are summarized in Table 1.

**Table 1:** Indicators of physical examination data.

Indicator	Range	Mean	Mean of diabetic population	Mean of non-diabetic population
Age	21–97	50	65	49
Gender	—	—	—	—
SH	—	—	—	—
AH	—	—	—	—
Ex. freq. (per week)	0–3	2	2	2
HDL-C	0.34–3.73	1.46	1.3	1.47
LDL-C	0.18–8.22	2.93	2.86	2.94
DBP	40–137	76.03	78.26	75.79
SBP	71–234	124.73	135.98	123.5
TG	0.19–23.95	1.44	2	1.38
HC	52–155	97.2	98.8	97.03
WC	31–161	84.06	90	83.41
Cr	13–361	69.51	72.31	69.2
NEU	0.4–17.84	3.48	3.96	3.43
LYM	0.34–155.13	2.04	2.14	2.03
WBC	1.55–218.45	6.06	6.72	5.99
UA	37–845	346.12	359.72	344.63
ECG	—	—	—	—
Ht	127–196.7	166.98	166.83	166.99
PLT	15–1,435	246.98	237.27	248.04
MONO	0.04–3.77	0.36	0.41	0.35
BUN	1.45–28.47	4.97	5.6	4.91
ALT	1–484	23.23	28.85	22.61
Wt	35.5–175.2	68.66	72.31	68.26
HR	45–128	73.25	74.29	73.14
EOS	0–3.94	0.15	0.17	0.14
RDW	30.9–79.4	41.45	41.86	41.4
BASO	0.01–13.61	0.037	0.04	0.036
P-LCR	7.1–70.5	27.04	27.32	27.01
MCV	56–118.8	89.8	90.29	89.75
MCH	14.5–49	30.13	30.367	30.1
BMI	14.36–53.36	24.53	25.9	24.38
WHtR	0.19–0.92	0.5	0.54	0.5

According to the World Health Organization’s guidelines on diagnostic methods for T2DM (35), the dataset was annotated. Data instances meeting either FBG ≥ 7.0 mmol/L or HbA1c ≥ 6.5% were classified as diabetic and labeled “1.” All other instances were classified as non-diabetic and labeled “0.” Consequently, each data entry in this dataset comprises 33 feature indicators and 1 outcome label.

From the original dataset, we screened records of individuals with three consecutive annual medical examinations, constructing a longitudinal dataset of 16,774 sequences. Among these, 14,997 sequences (89.4%) had a non-diabetic outcome (label “0”) in the third year, 1,777 sequences (10.6%) had a diabetic outcome (label “1”) in the third year. This dataset exhibits severe class imbalance, with diabetic samples (the minority class) significantly outnumbered by non-diabetic samples (the majority class) at an approximate ratio of 1:8.34. Such imbalance risks biasing the model toward the majority class, thereby degrading predictive performance for the minority class. To enhance the reliability of model evaluation and reduce randomness arising from training and testing set partitioning, A 5-fold stratified cross-validation scheme (36) was adopted for model training and evaluation. The full dataset was split into five subsets via stratified sampling, maintaining consistent positive–negative sample distribution across all subsets as in the original dataset. The sample distribution of each fold experiment is shown in Table 2.

**Table 2:** Sample distribution of each fold experiment.

Fold	Training Set	Positive (in training set)	Negative (in training set)	Test set	Positive (in test set)	Negative (in test set)
Fold 1/5	13,419	1,407	12,012	3,355	370	2,985
Fold 2/5	13,419	1,434	11,985	3,355	343	3,012
Fold 3/5	13,419	1,426	11,993	3,355	351	3,004
Fold 4/5	13,419	1,420	11,999	3,355	357	2,998
Fold 5/5	13,420	1,421	11,999	3,354	356	2,998

### 2.2 Data preprocessing

Data preprocessing serves two primary purposes: (1) standardizing data formats to ensure compatibility with model training and analysis, and (2) enhancing data quality to improve model predictive performance. Key preprocessing steps include data cleaning, data conversion, and feature map construction.

#### 2.2.1 Data cleaning

Data cleaning refers to the processing of missing and abnormal values of physical examination indicators. Due to variations in medical examination packages selected by individuals across years, certain indicators may be missing from specific years’ records. Given the limited missing data and the absence of significant skewed distributions or extreme values in the dataset, we employed mean imputation method to replace missing values with either the individual’s mean for that indicator across other years or the overall indicator mean value. This method is simple and preserves data integrity without introducing distributional bias. We use the same method to processing the abnormal value.

#### 2.2.2 Data conversion

Data conversion involves encoding categorical (non-numeric) features and normalizing numerical features. For categorical indicators, we employed one-hot encoding to convert each category into a binary value. Table 3 outlines the encoding rules for all categorical indicators.

**Table 3:** Encoding rules for non-numeric indicators.

Feature	Encoding rule
ECG	Abnormal: 1, normal: 0
SH	With smoke history: 1, without: 0
AH	With alcohol history: 1, without: 0
Gender	Male: 1, female: 0
Ex. freq. (per week)	None: 0, 1–2 times: 1, 3 times: 2, > 3 times: 3

For numerical indicators, we applied Min-Max Normalization to linearly scale each feature to the [0, 1] interval while preserving the original data distribution. This step helps to mitigate scale discrepancies and ensures balanced contributions of different features to the model. The normalization formula is defined as:


$$X_{\text{scaled}}=\frac{X-X_{\min}}{X_{\max}-X_{\min}}$$

where: *X* denotes raw indicator value, *X*_min_ is minimum value of the indicator, *X*_max_ is maximum value of the indicator.

#### 2.2.3 Feature map

**Figure 1:**
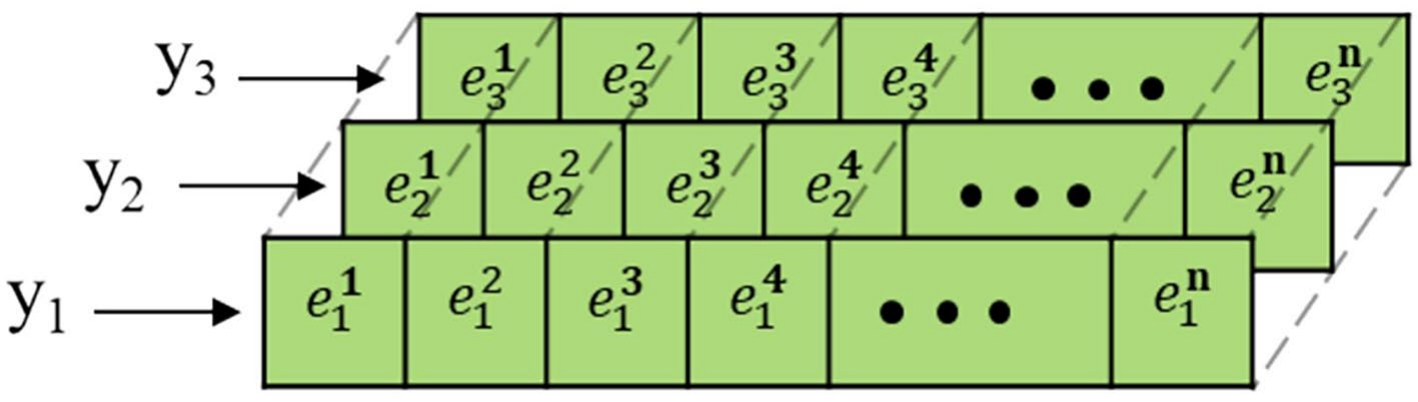
Feature map constructed based on physical examination data series.

To predict T2DM by using physical examination data series, we organized the examination sequences into a feature map format as the input for our proposed model. For a single patient with continuous *m*-year examination records, the data were structured into a 1 × *n* × *m* feature map, Figure 1 illustrates the construction of a feature map using 3 years of sequential examination data, where: *n* represents the number of clinical indicators per examination record. In this study, *n* was set to 33 when no feature selection was applied. *y_i_* denotes the examination data for the *i_th_* year. $e_{i}^{j}$ indicates the value of the *j_th_* clinical indicator in the *y_i_* record.

### 2.3 Prediction model based on physical examination data series

**Figure 2:**
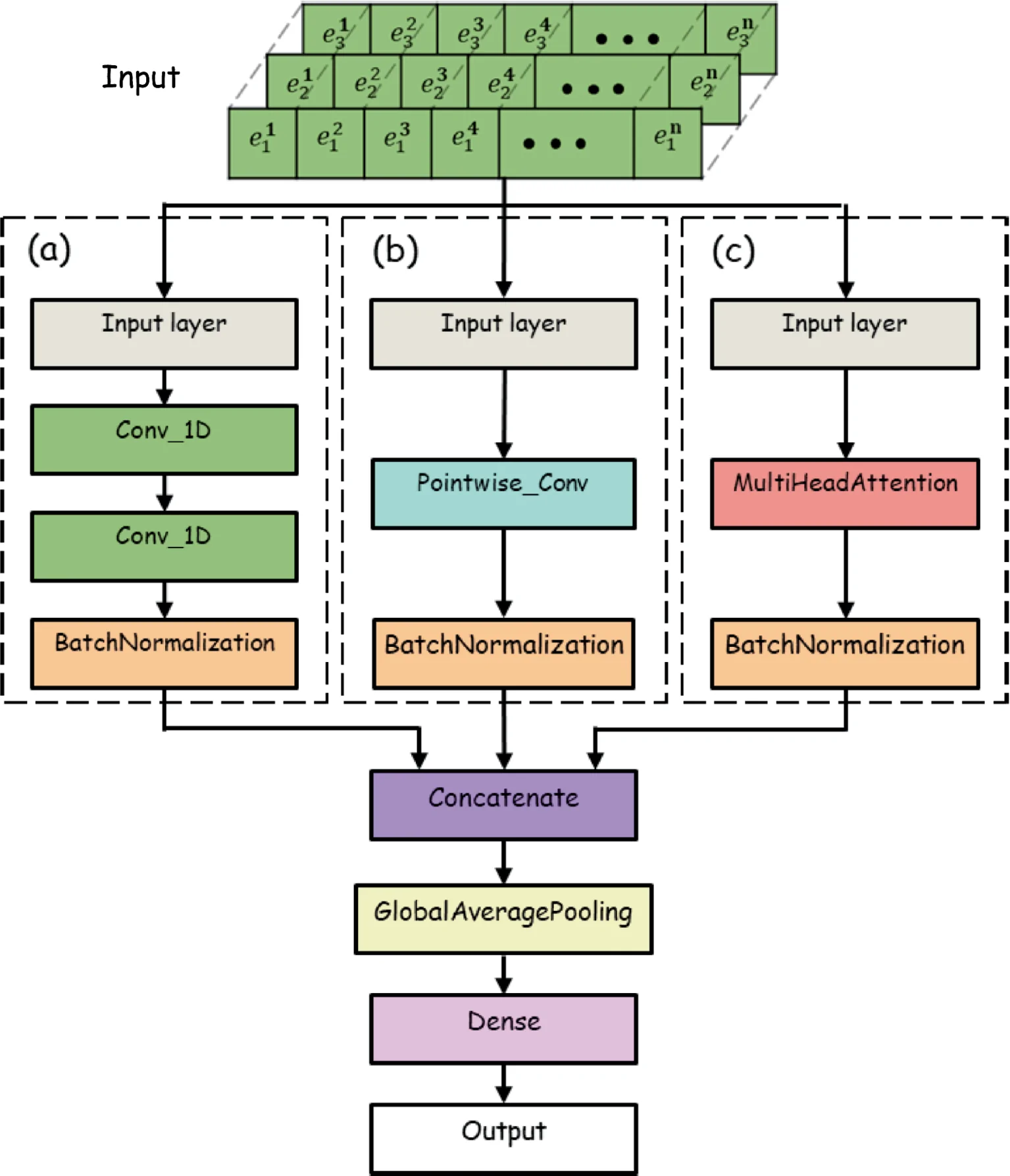
Structure of the MFFNet.

As illustrated in Figure 2, we propose a T2DM prediction model (MFFNet) based on the physical examination data series. MFFNet integrates three specialized feature extraction branches to capture diabetes-predictive information: (1) Horizontal branch (branch a): Extracts features from cross-sectional data *y_i_* (e.g., annual examination indicators) to analyze their impact on prediction outcomes. We hypothesize that inter-indicator relationships within the same time period influence diabetes risk, and employ Conv_1D (1D convolution) to capture these patterns. (2) Longitudinal branch (branch b): Extracts temporal dynamics of specific clinical indicator $e_{i}^{j}$ (e.g., longitudinal trajectories of Creatinine) using Pointwise_Conv (pointwise convolution), reflecting how individual metrics evolve over time. (3) Cross Branch (Branch c): Captures cross-indicators temporal dependencies (e.g., interactions between HbA1c and Waist Circumference across years) through multi-head attention mechanism of Transformer (37), which effectively models complex inter-feature relationships and non-linear interactions. Finally, the features extracted from the three branches are fused to predict T2DM. MFFNet comprehensively extracts and integrates feature information from three dimensions (horizontal, longitudinal, and cross-feature interactions) within physical examination data series, enabling more effective mining of latent patterns in the data. In addition, the BatchNormalization layer in MFFNet is used to accelerate the training process and improve its generalization ability. The GlobalAveragePooling layer is used to pool features globally, reducing data dimensions while retaining the most important feature information. The final Dense layers is used to generate the final probability of diabetes prediction results. Table 4 shows the structure and parameter detail of MFFNet. Algorithm 1 shows the pseudocode of MFFNet. The complete implementation code of MFFNet has been publicly released on GitHub at https://github.com/sunbijun1017/my-shiyan-project and on Hugging Face at https://huggingface.co/spaces/sunbijun1017/my-shiyan-space/tree/main.

**ALGORITHM 1:**
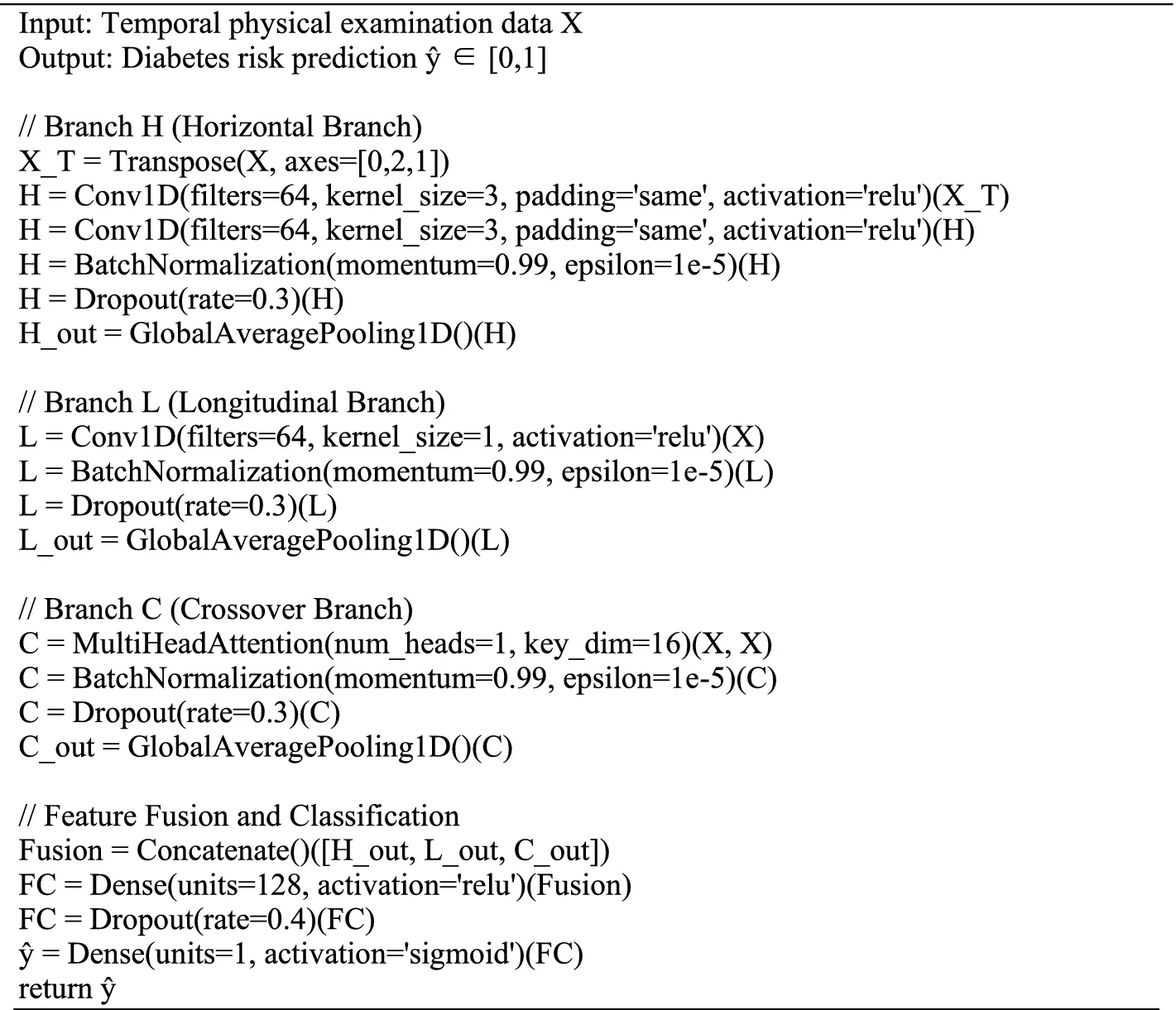
The pseudocode of MFFNet.

**Table 4:** Parameters of the MFFNet.

Module	Layer	Filter Size	Filter number	Other parameters
Branch_H	Conv_1D	1 × 3	64	activation = ‘relu’
	Conv_1D	1 × 3	64	activation = ‘relu’
	BatchNormalization	—	—	momentum = 0.99, epsilon = 1e-5
Branch_L	Pointwise_Conv	1 × 1	64	activation = ‘relu’
	BatchNormalization	—	—	momentum = 0.99, epsilon = 1e-5
Branch_C	Multi-head Attention	—	—	num_heads = 1, key_dim = 16
	BatchNormalization	—	—	momentum = 0.99, epsilon = 1e-5
Feature fusion	Concatenate	—	—	axis = −1
Down sampling	Global AveragePooling	—	—	data_format = ‘channels_last’, keepdims = False
Full connection	Dense	—	—	units = 128, activation = ‘relu’
Output	Dense	—	—	units = 1, activation = ‘sigmoid’

## 3 Results

### 3.1 Experimental setting

The experimental environment in this paper is Ubuntu 18.04 LTS 64 bit operating system, with Intel Xeon Bronze 3,106, 1.7GHz × 16 core CPU, 64GB of system memory, Nvidia GeForce GTX1080Ti × 2 GPU, 12GB of memory per block, and Pytorch deep learning framework. The main hardware cost comes from the workstation equipped with the dedicated GPU, costing approximately 40,000 RMB. All software tools adopted in this study are open-source and thus free of charge. During the model training process, Weighted Binary Cross Entropy Loss (38) is introduced to dynamically adjust the loss weights for different types of samples, enabling the network to focus more on hard-to-classify samples. The batch size is set to 32 and the epoch is set to 100. Simultaneously, we use Adam optimizer to dynamically adjust the learning rate of each parameter, to accelerate training and optimize model performance. We Use Dropout layers to randomly shut down a portion of neurons during training to improve the model’s generalization ability.

### 3.2 Evaluation metrics and evaluation methods

To comprehensively evaluate model performance, Sensitivity (Recall), Specificity, PPV (Positive Predictive Value, or Precision), NPV (Negative Predictive Value), F1 Score, ROC_AUC, PR-AUC and Brier Score are used as evaluation metrics in Table.5. These metrics are calculated from the confusion matrix.

**Table 5:** Metrics for evaluating the model performance.

Metric	Function
Sensitivity	Reflects the model’s ability to identify positive (diabetic) samples
Specificity	Reflects the model’s ability to identify negative (non-diabetic) samples
PPV	Reflects the reliability of the model’s positive predictions
NPV	Reflects the reliability of the model’s negative predictions
F1 score	Comprehensively evaluates the model’s precision and sensitivity, balancing their performance
ROC-AUC	Reflects the model’s overall ability to distinguish positive and negative samples; value closer to 1 indicates stronger discrimination
PR-AUC	Reflects the model’s comprehensive precision-recall performance across thresholds; more informative for imbalanced samples
Brier score	Measures probabilistic prediction accuracy; lower values indicate better calibration

Sensitivity is defined as the proportion of samples correctly predicted to have diabetes among all samples labeled as having diabetes. The calculation formula is as follows:


$$\text{Sensitivity}=\frac{\mathrm{TP}}{\mathrm{TP}+\mathrm{FN}}$$

Specificity refers to the proportion of samples correctly predicted non-diabetic among all actual non-diabetic samples.


$$\text{Specificity}=\frac{\mathrm{TN}}{\mathrm{TN}+\mathrm{FP}}$$

PPV is the proportion of samples correctly predicted to have diabetes out of all samples predicted to have diabetes. The calculation formula is as follows:


$$\mathrm{PPV}=\frac{\mathrm{TP}}{\mathrm{TP}+\mathrm{FP}}$$

NPV is the proportion of correctly predicted non-diabetic samples in all predicted non-diabetic samples. The calculation formula is as follows:


$$\mathrm{NPV}=\frac{\mathrm{TN}}{\mathrm{TN}+\mathrm{FN}}$$

The F1 score comprehensively evaluates the importance of Precision and Recall metrics, calculated as follows:


$$F1=2\times \frac{\text{Precision}\times \text{Recall}}{\text{Precision}+\text{Recall}}$$

In above formulas, TP = true positive, FP = false positive, FN = false negative, TN = true negative.

### 3.3 Model performance evaluation for detecting T2DM

**Figure 3:**
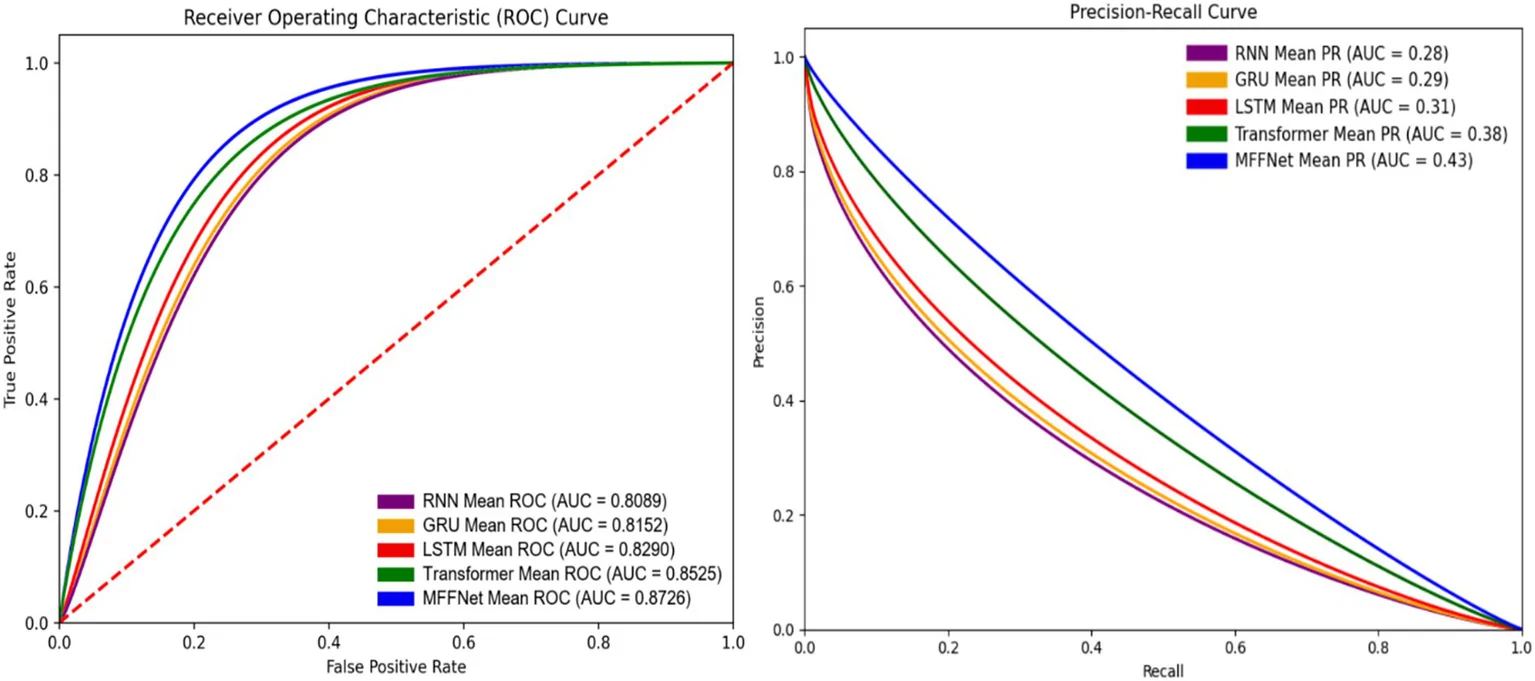
ROC curves and precision-recall curves for various models.

To validate the effectiveness of the MFFNet model together with time-series data in detecting T2DM, the model was experimentally compared with classical machine learning models. Four state-of-the-art sequential models were selected for comparison: RNN, LSTM, GRU, and Transformer. The evaluation metrics, including Sensitivity, Specificity, NPV, PPV, F1 score, ROC-AUC, PR-AUC, Brier score, for each model are presented in Table 6. Each metric value is reported as the mean calculated from 5-fold cross-validation experiments. It is evident that all models demonstrated good discriminative capacity for diabetes detection under such extremely imbalanced sample distribution, confirming the predictive value of longitudinal data. Among these models, MFFNet demonstrated the best performance, with most metrics reaching the highest values. Specifically, MFFNet demonstrated high sensitivity (Sensitivity = 0.8766) and negative predictive value (NPV = 0.9799), indicating a low missed diagnosis rate in identifying potential diabetic patients and confirming its efficacy in screening high-risk populations. This suggests that the model holds significant potential as an initial screening tool in clinical practice. However, the positive predictive value (PPV = 0.2650) was relatively low, suggesting that a substantial proportion of individuals identified as positive were actually non-diseased, resulting in a high false-positive rate. This phenomenon is closely associated with the large proportion of negative samples and the low prevalence of T2DM within the dataset. Furthermore, the Specificity (0.7082) was moderate, and the F1 score (0.4058) was constrained by the low PPV, reflecting limitations in the model’s precision. The Brier score of 0.1613 indicated that the probabilistic predictions were reasonably calibrated without systematic bias. Figure 3 displays the ROC curves and the Precision-Recall curves for the five models. Based on the AUC values, the discriminative capabilities for diabetes prediction rank as follows: MFFNet > Transformer > LSTM > GRU > RNN. Table 6 also presents the inference time per sample and memory consumption during inference for each model. Obviously, MFFNet achieves the shortest inference time of 52.045 ms, while its memory usage is comparable to that of other models at 0.110 MB. In conclusion, MFFNet is more suitable for large-scale population screening and early risk warning​ rather than the other time-series models.

**Table 6:** Comparison results of MFFNet and other models.

Model	Sensitivity	Specificity	NPV	PPV	F1	ROC AUC	PR-AUC	Brier	Inference Time (ms/sample)	Peak Memory Usage (MB)
RNN	0.8050	0.6995	0.9684	0.2414	0.3709	0.8089	0.2826	0.1783	69.459	0.105
GRU	0.8191	0.6831	0.9705	0.2350	0.3643	0.8152	0.2929	0.1856	66.740	0.107
LSTM	0.8278	0.6990	0.9721	0.2465	0.3795	0.8290	0.3145	0.1775	63.606	0.104
Transformer	0.8285	0.7226	0.9733	0.2642	0.3995	0.8525	0.3759	0.1831	65.202	0.110
**MFFNet**	**0.8766**	**0.7082**	**0.9799**	**0.2650**	**0.4058**	**0.8726**	**0.4322**	**0.1613**	**52.045**	**0.110**

The bold line is the most effective.

### 3.4 Ablation experiment

MFFNet comprises three branch modules: the horizontal branch (H-Branch), the longitudinal branch (L-Branch), and the cross branch (C-Branch). To validate the effectiveness of each branch in predicting T2DM, a model ablation experiment was conducted, with the results presented in Table 7. The findings clearly indicate that information extracted from all three branches plays a critical role in diabetes prediction. Feature information derived from any single branch yielded satisfactory results. Notably, utilizing cross-sectional features alone yielded the best prediction performance. This demonstrates the paramount importance of cross-sectional features in diabetes prediction. Combining features from any two branches further enhanced prediction performance, confirming that both longitudinal temporal features and crossover features contribute valuable information. The fusion of features from all three branches resulted in the highest predictive performance.

**Table 7:** Results of ablation experiment.

H-Branch	C-Branch	L-Branch	Sensitivity	Specificity	NPV	PPV	F1	ROC- AUC	PR-AUC	Brier
√	×	×	0.8153	0.6549	0.9674	0.2212	0.3472	0.8035	0.3176	0.1882
×	√	×	0.8352	0.7018	0.9744	0.2564	0.3896	0.8410	0.3625	0.1970
×	×	√	0.8103	0.6679	0.9678	0.2268	0.3531	0.8058	0.3207	0.1859
√	√	×	0.8511	0.6915	0.9759	0.2494	0.3842	0.8479	0.3620	0.1811
√	×	√	0.8126	0.7198	0.9708	0.2592	0.3912	0.8425	0.3563	0.1750
×	√	√	0.8529	0.6905	0.9757	0.2470	0.3826	0.8463	0.3613	0.1810
√	√	√	**0.8766**	**0.7082**	**0.9799**	**0.2650**	**0.4058**	**0.8726**	**0.4322**	**0.1613**

The bold line is the most effective.

### 3.5 Model performance evaluation for predicting T2DM

We further employed MFFNet to predict the onset risk of type 2 diabetes mellitus (T2DM) within the subsequent 1 year. Limited by the available dataset, we adopted physical examination data from two consecutive years (year-1 and year-2) as the model input, and the T2DM screening results of the third year were taken as the output label for model training. Four classic time-series models, including RNN, LSTM, GRU and Transformer, were selected as comparative baselines. The experimental results are presented in Table 8. It can be clearly observed that MFFNet outperforms all comparative models across all evaluation metrics, demonstrating that MFFNet is more suitable for T2DM risk prediction than other time-series models.

**Table 8:** Results for predicting T2DM by various time-series models.

Model	Sensitivity	Specificity	NPV	PPV	F1	ROC- AUC	PR-AUC	Brier
RNN	0.7514	0.7141	0.9607	0.2380	0.3610	0.7916	0.2601	0.1939
GRU	0.7870	0.6950	0.9705	0.2370	0.3624	0.8057	0.2848	0.1927
LSTM	0.7829	0.7111	0.9694	0.2495	0.3789	0.8179	0.3068	0.1816
Transformer	0.7940	0.7486	0.9685	0.2724	0.4054	0.8477	0.3661	0.1877
MFFNet	0.8035	0.7640	0.9711	0.2916	0.4253	0.8648	0.4109	0.1691

### 3.6 SHAP-based interpretability analysis of MFFNet

**Figure 4:**
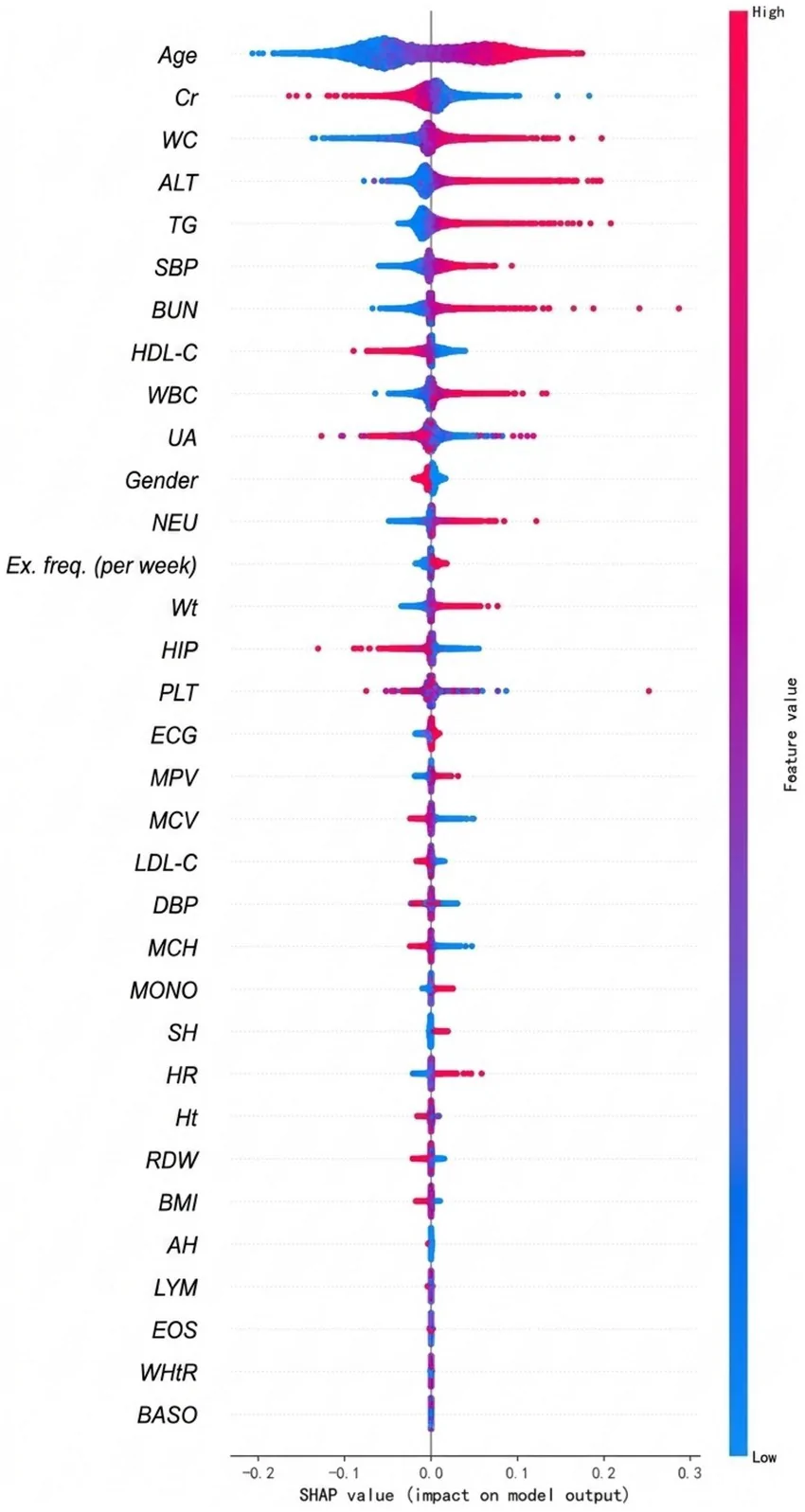
SHAP values of each feature.

Figure 4 illustrates the global feature importance analysis based on SHAP values. As depicted in the figure, MFFNet predominantly relies on features such as Age, Cr, WC, ALT, TG, and SBP, which exhibit broad distributions of SHAP values and thus significantly influence model outputs. Notably, Age demonstrates the widest span of SHAP values, with high values shifting markedly to the right, indicating a substantial increase in predicted diabetes risk with advancing age. This observation aligns with established epidemiological evidence identifying age as an independent risk factor for diabetes. Furthermore, metabolic and anthropometric indicators, including WC, TG, and Weight, show considerable positive contributions, highlighting the critical role of central obesity and dyslipidemia in the pathogenesis of diabetes within this cohort. In contrast, hematological parameters such as BASO, WHtR, and LYM cluster tightly around zero with no discernible pattern, suggesting their marginal contribution to the model’s predictions. These findings imply that simplifying models by excluding such low-impact features could effectively reduce complexity and enhance interpretability in future modeling. Regarding feature directionality, the majority of high feature values correspond to positive SHAP values, indicating that states characterized by advanced age, elevated creatinine, hypertension, and hyperlipidemia tend to increase the predicted probability of disease. Conversely, low feature values are mapped to negative SHAP values, reflecting the intuitive medical rationale that normal or low levels of these metrics are conducive to risk reduction. Overall, the risk patterns learned by the model are highly consistent with classical pathophysiological mechanisms of T2DM.

## 4 Discussion

Literature review indicates that current research on T2DM classification primarily relies on traditional machine learning methods and static cross-sectional data. We propose that longitudinal physical examination data can reveal dynamic temporal evolution patterns of indicators that static data cannot obtain, and this temporal variation information reflects, to some extent, the gradual progression of disease development. Therefore, time series data analysis is more suitable for early detection and risk prediction of diabetes than static data.

To validate our idea, we employ various traditional machine learning algorithms combined with single-year static physical examination data to construct T2DM detection models, which are then compared against the time-series models in terms of prediction performance. The benchmark algorithms selected include K-Nearest Neighbors (KNN), Random Forest (RF), Support Vector Machine (SVM), and Gradient Boosting Decision Tree (GBDT). Experimental results are presented in Table 9. By comparing these with the predictive performance of the time-series model shown in Table 6, it is evident that detection models based on traditional machine learning and static data exhibit suboptimal overall performance, with all evaluation metrics being markedly lower than those of conventional time-series models and our proposed MFFNet in this study.

**Table 9:** Results of various models for static T2DM classification.

Model	Sensitivity	Specificity	NPV	PPV	F1	ROC AUC	PR-AUC	Brier
KNN	0.7953	0.7273	0.9702	0.2413	0.3702	0.8014	0.3346	0.0733
RF	0.7912	0.7657	0.9711	0.2691	0.4016	0.8520	0.3529	0.1588
GBDT	0.7969	0.7362	0.9708	0.2477	0.3780	0.8418	0.3253	0.0756
SVM	0.8026	0.7114	0.9706	0.2326	0.3607	0.8274	0.3764	0.0789

Both cross-sectional and longitudinal experimental results indicate that the models exhibit limited classification accuracy for positive cases, the highest PPV achieved by longitudinal and cross-sectional experiments are 0.2916 and 0.2691, respectively, This phenomenon could be primarily attributed to two factors: (1) the severe class imbalance (1:8.34) biases the model toward prioritizing sensitivity at the expense of precision; (2) the substantial phenotypic overlap between diabetes patients and high-risk populations, such as those with prediabetes, impaired glucose tolerance, and metabolic syndrome, makes it difficult for the model to delineate precise decision boundaries. In future work, we will continuously collect more physical examination data series, and contrast a more accurate and robust classification model based on a balanced dataset.

To verify that the model captures the progression of T2DM rather than confounding signals of systemic health deterioration, we split the dataset into two age-based subgroups. The younger subgroup consists of samples under 60 years old, containing 35,921 records with 11,943 positive cases. The older subgroup includes samples aged 60 years and above, containing 14,159 records with 4,710 positive cases. Table 10 presents the T2DM classification results for both subgroups. For the younger group (<60 years), the model achieves significantly higher ROC-AUC (0.8819), sensitivity (0.8009), and specificity (0.7990) compared to the older group (≥60 years), which shows a ROC-AUC of 0.7245, sensitivity of 0.7014, and specificity of 0.6198. Additionally, the younger group exhibits a lower Brier score (0.1280 vs. 0.2191). These findings indicate that the proposed model demonstrates stronger discriminative ability in younger populations. This performance gap likely arises from the relatively homogeneous physiological status and physical characteristics within younger cohorts, where interference from non-diabetic confounders is minimal. Consequently, the model can accurately extract metabolic signatures specific to T2DM progression. In contrast, older individuals often experience multimorbidity and diverse age-related physiological changes, such as sarcopenia, chronic inflammation, and progressive multi-organ dysfunction. Under these conditions, confounding signals reflecting systemic health deterioration become highly intertwined with authentic T2DM progression features, introducing substantial noise that ultimately impairs model performance in older subjects. This observation further confirms that the model’s predictions are not dominated by non-specific aging-related deterioration signals. Moreover, the younger subgroup suffers from severe class imbalance due to an overwhelming number of healthy negative samples (total = 12,035, positive = 562, negative = 11,473), which leads to a low PPV (0.1754) and an F1-score (0.2821). In contrast, the older cohort has a more balanced class distribution (total = 4,739, positive = 1,215, negative = 3,524), resulting in higher PPV (0.3988) and F1-score (0.4996).

**Table 10:** T2DM classification results in younger and older subgroups.

Age	Sensitivity	Specificity	NPV	PPV	F1	ROC- AUC	PR-AUC	Brier
<60	0.8009	0.7990	0.9885	0.1754	0.2821	0.8819	0.3039	0.1280
> = 60	0.7014	0.6198	0.8622	0.3988	0.4996	0.7245	0.4703	0.2191

**Figure 5:**
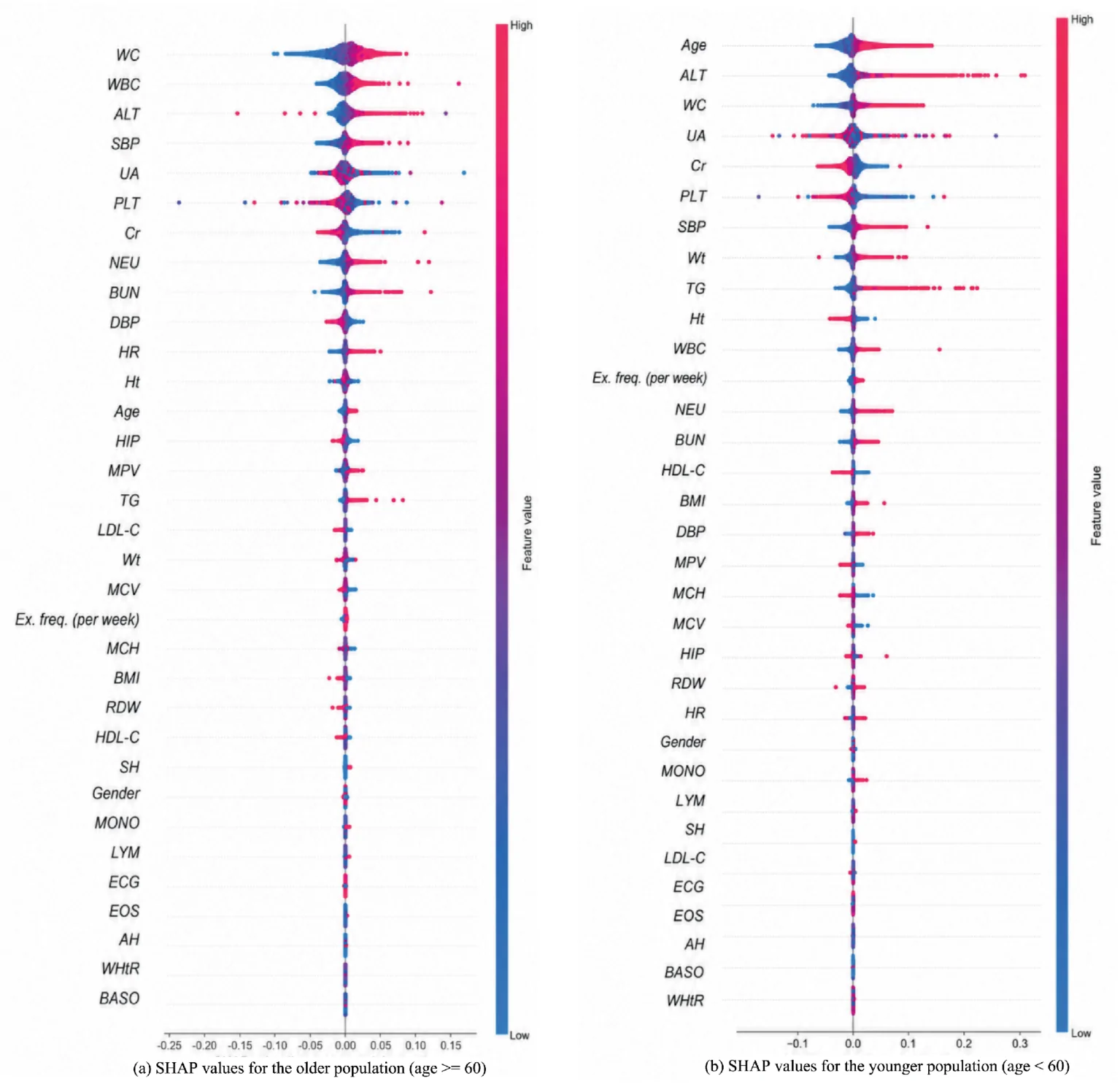
SHAP values of each feature for younger and older populations. **(a)** SHAP values for the older population **(b)** SHAP values for the younger population.

Furthermore, Figure 5 presents SHAP beeswarm plots for both young and older populations. Core predictive features, including WC and ALT, consistently rank highest in importance across both groups. These features are canonical pathological markers of T2DM rather than general indicators of aging-related health deterioration. Notably, age is the most influential predictor only among young participants, while its predictive weight declines sharply in the older populations. The consistent direction of contribution from key clinical indicators across distinct age cohorts demonstrates that our model primarily captures T2DM-specific progression patterns rather than confounding aging signals, thereby maintaining stable predictive logic across populations with diverse ages.

## 5 Limitations

Although our work has demonstrated superior performance in diabetes classification and dynamic diabetes prediction compared to static methods, several limitations were identified during the experimental process. First, regarding data resources, the source data in this study exhibited a significant imbalance between positive and negative samples, and the data quality is suboptimal, containing noise and outliers. These issues present considerable challenges in training a model with high accuracy and robust performance. Additionally, the sample size is limited to the collected physical examination sequence data, which may constrain the model’s generalizability. To enhance robustness and generalization, it is necessary to collect data spanning more consecutive years and from a broader range of sources for further training and validation. Moreover, due to the particularity of continuous longitudinal physical examination sequence data, we were unable to find similar publicly available datasets or related studies through extensive literature review and public database searches, making direct comparative experiments with similar studies on the same or highly comparable datasets infeasible. Second, in terms of model design, the proposed model primarily focuses on the temporal correlations within sequence data and does not fully integrate other multimodal data that may influence diabetes onset, such as clinical symptoms, additional lifestyle factors, and genetic information. Incorporating multimodal data could further enhance prediction accuracy and comprehensiveness, representing a key direction for future optimization. Third, the interpretability of the model requires further improvement. Although the model achieves good predictive performance, the internal mechanisms by which it identifies key physical examination indicators and temporal changes to predict diabetes have not been fully elucidated. Enhancing model interpretability will facilitate greater acceptance among medical professionals and promote its practical application in clinical settings. Forth, The AI model serves only as a pre-screening too1. Positive predictions should trigger confirmatory tests (e.g., OGTT) and alert early interventions. Due to class imbalance, a large number of positive predictions are false positives, which will result in numerous unnecessary OGTT tests. This not only raises medical costs and consumes clinical resources, but also causes anxiety and time costs for patients. Considering this limitation, we clearly position the model as a pre-screening tool for general populations. It screens out potential high-risk individuals efficiently. Compared with universal OGTT testing, the combination of AI screening and secondary confirmation effectively optimizes resource allocation and controls overall medical burden in routine clinical scenarios.

## 6 Conclusion

This paper proposes MFFNet, a multi-dimensional feature extraction and fusion architecture built upon longitudinal physical examination data sequences, to tackle the challenges of type 2 diabetes mellitus (T2DM) classification and early risk prediction. MFFNet extracts and integrates information across three complementary dimensions—horizontal, longitudinal, and cross-dimensional—from input feature maps, supporting accurate T2DM detection and dynamic early risk warning. Validated on a 3-year longitudinal physical examination dataset from the Health Management Center of Beijing Hospital, MFFNet delivers strong T2DM classification performance under an extremely imbalanced class distribution (positive:negative = 1:8.34), achieving a sensitivity of 0.8766, specificity of 0.7082, negative predictive value (NPV) of 0.9799, and precision–recall AUC (PR-AUC) of 0.4322. These results surpass those of state-of-the-art time-series models including RNN, LSTM, GRU, and Transformer. Furthermore, a SHAP-based interpretability analysis was performed, revealing that age, creatinine (Cr), waist circumference (WC), alanine transaminase (ALT), triglycerides (TG), and systolic blood pressure (SBP) serve as the most critical features for T2DM detection by MFFNet, whereas basophils (BASO), waist-to-height ratio (WHtR), eosinophils (EOS), lymphocytes (LYM), and alcohol history (AH) contribute marginally to model predictions. Comprehensive experimental results demonstrate that the MFFNet framework, combined with longitudinal physical examination data, outperforms both static cross-section-based prediction and conventional time-series-based dynamic prediction for T2DM. To address the limitations observed in this study, we will continue to collect continuous longitudinal physical examination sequence data, expand the sample size and enhance data diversity, and focus on investigating the following two issues: first, Constructing a more balanced dataset to improve the model’s accuracy on positive samples; second, integrating multi-modal data (e.g., lifestyle factors, clinical symptoms, and genetic information) to optimize model architecture, thus further enhancing prediction accuracy and generalization capability.

## Data Availability

The data that support the findings of this study are available on request from the corresponding author. The data are not publicly available due to privacy or ethical restrictions.
